# Supplementary material for: Anticoagulant Properties of Coated Fe-Pd Ferromagnetic Shape Memory Ribbons
Source: Int J Mol Sci. 2023 Jan 26;24(3):2452. doi: 10.3390/ijms24032452 (PMC9917221; doi:10.3390/ijms24032452)
Supplement: Supplementary file 1 [file ijms-24-02452-s001.zip › ijms-2070157-supplementary.pdf]

Supporting Information for:

Anticoagulant properties of coated Fe-Pd ferromagnetic shape memory ribbons

Alexander Bunge[1], Alexandru Chiriac[2], Mihaela Sofronie[3], Izabell Crăciunescu[1], Alin Sebastian Porav[1] and Rodica Turcu[1*]

[1] National Institute R&D for Isotopic and Molecular Technology, 67-103 Donat Street, Cluj-Napoca 400293, Romania; alexander.bunge@itim-cj.ro; izabell.craciunescu@itim-cj.ro; sebastian.porav@itim-cj.ro

[2] "Grigore T. Popa" University of Medicine and Pharmacy, Str. Universității nr. 16, Iasi 700115, Romania 2; alexandru.chiriac@umfiasi.ro

[3] National Institute of Materials Physics, Atomistilor Street 405A, Bucharest-Magurele 077125, Romania; mihsof@infim.ro

* National Institute R&D for Isotopic and Molecular Technology, 67-103 Donat Street, Cluj-Napoca 400293, Romania; rodica.turcu@itim-cj.ro

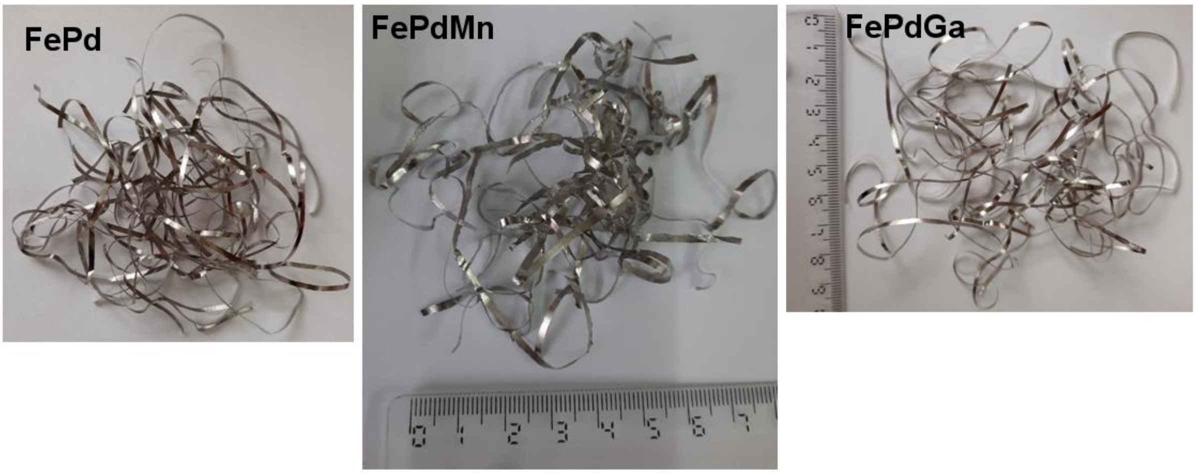

Figure S1: Pictures of FePd, FePdMn and FePdGa ribbons before thermal treatment.

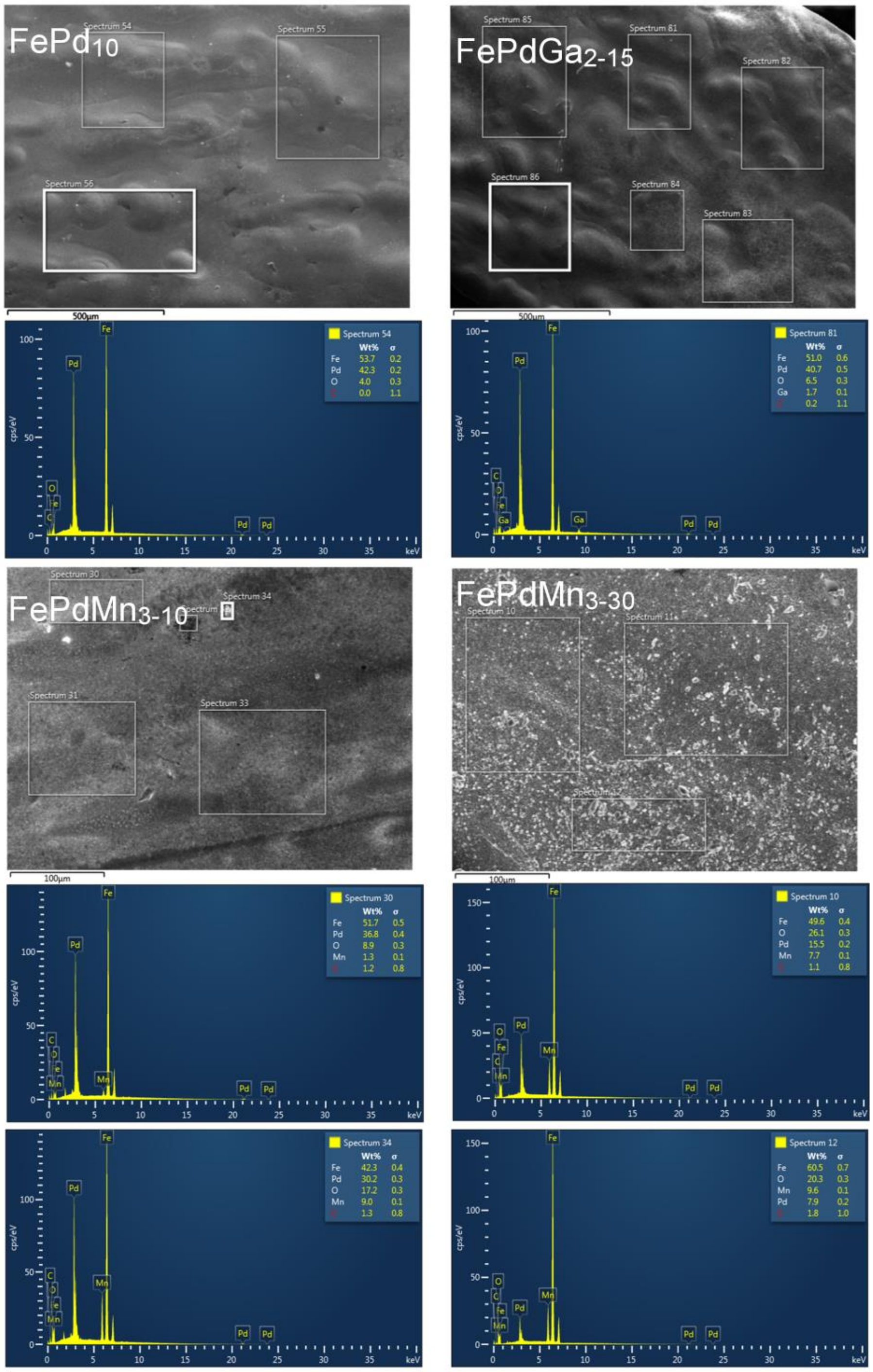

Figure S2. EDX of the uncoated FSMA ribbons

Potassium rhodizonate was prepared according to[1]: Inositol (10 g) was heated with nitric acid (69%, 25 mL) to reflux for 3 hours, until the brown gas evolution ceased. Afterward, to the solution was added water to 100 mL, acetic acid (50 mL) and while cooling in an ice bath, KOH (40 g). After stirring over night under air, the precipitate was filtered, washed with isopropanol, and dried in an oven to yield potassium rhodizonate as a brownish powder.

The determination of sulfate in sulfated pectins 2 was done similar to [2]:

Solution A (Ba-buffer) was prepared from 5 mL of 2 M acetic acid, 1 mL of 0.01 M $BaCl_2$ solution, and 4 mL of 0.05 M sodium hydrogen carbonate solution, diluted with EtOH to 150 mL.

Solution B (rhodizonate) was prepared by dissolving 6 mg potassium rhodizonate, 113 mg sodium ascorbate and 505 µL 1M HCl in 20 mL of water, then diluting to 100 mL with EtOH, leaving it stand for 15 min and then storing at -20 °C.

The sulfate stock solution for calibration was a 0.400 mM sodium sulfate solution.

To approximately 10.0 mg of sulfated pectin was added 10 µL of 40% NaOH solution in a glass test tube, the test tube put in the oven at 130 °C until dry and heated with a burner for ca. 6 seconds (the glass was glowing slightly red). After cooling down, water (3.000 mL) were added, an aliquot diluted with water appropriately, and 200 µl of that solution mixed with 1200 µl of solution A and 600 µL of solution B, reacted for 10 min and the adsorption at 520 nm compared to the calibration curve.

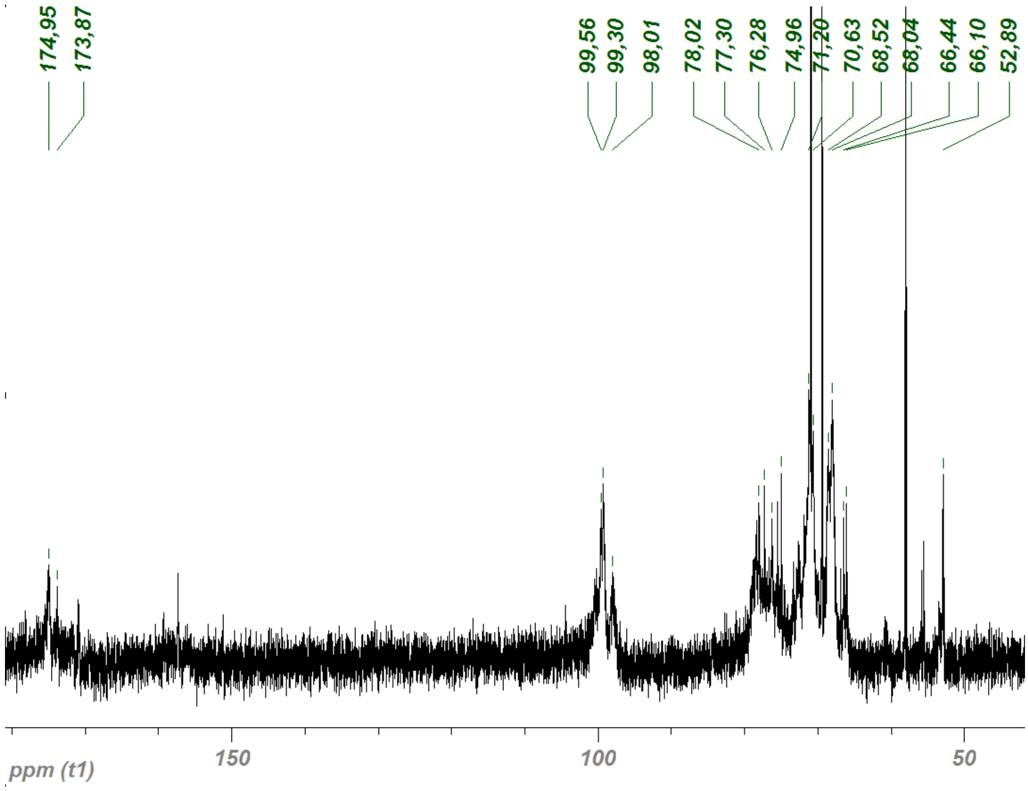

Figure S3: ^{13}C NMR of **2b**

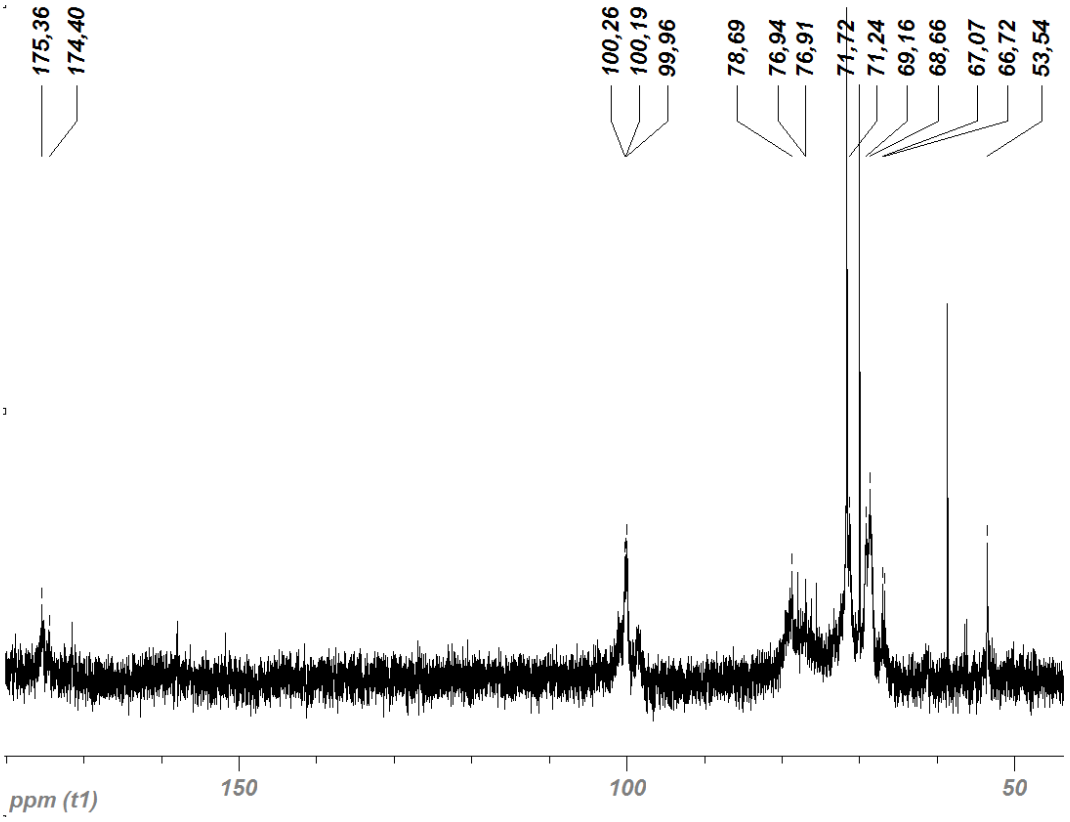

Figure S4: ^{13}C NMR of **2c**

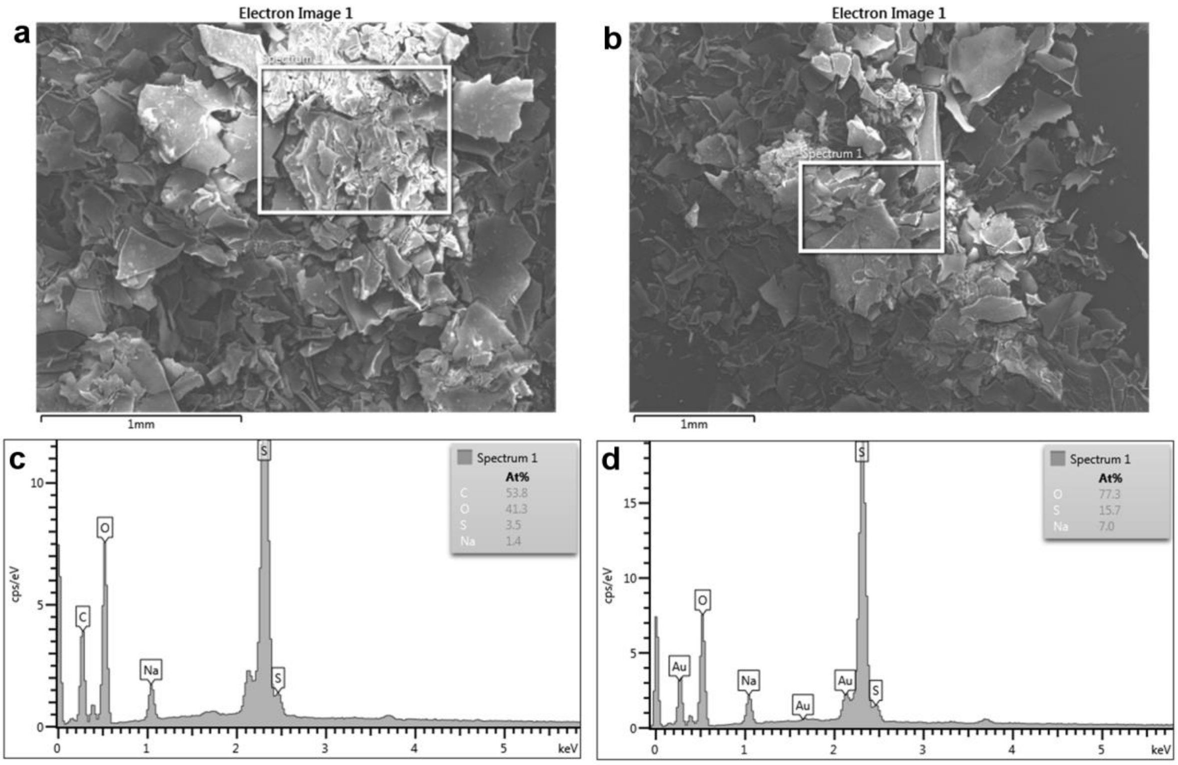

Figure S5: SEM and EDX of **2b** (a, c) and **2c** (b, d)

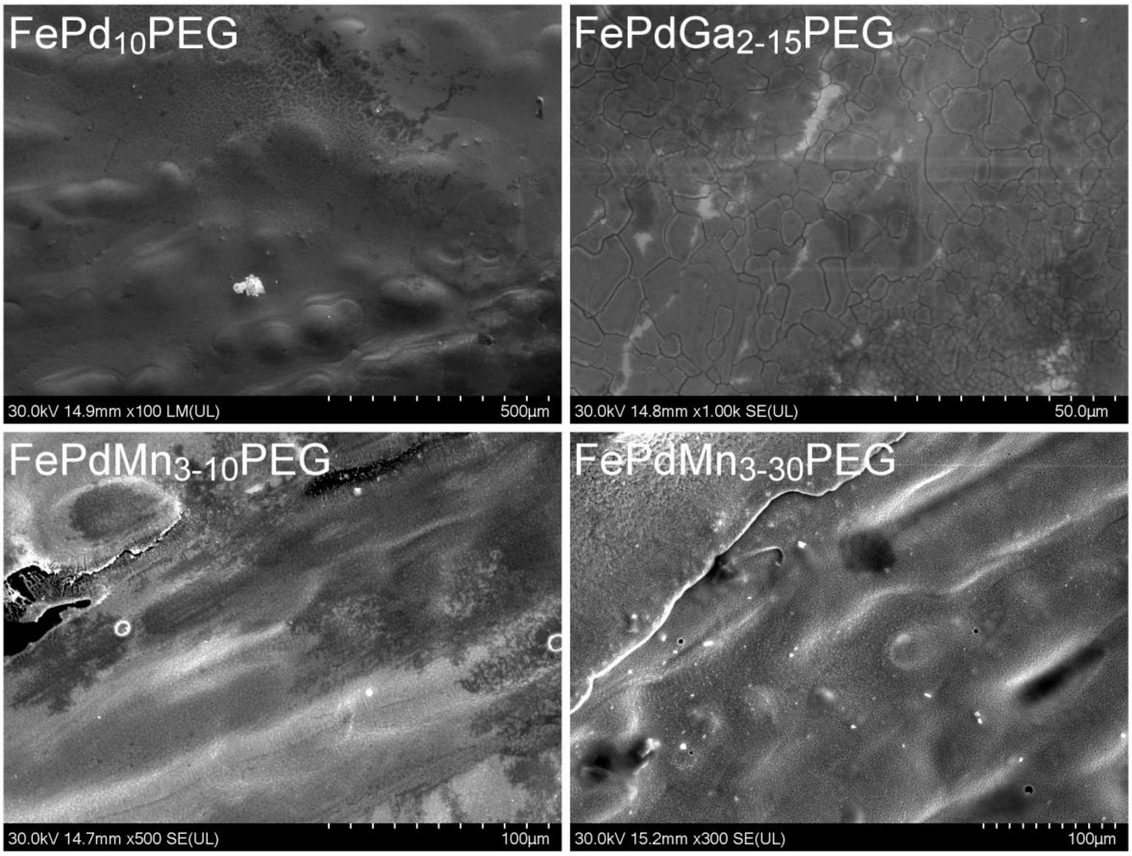

Figure S6: SEM of PEG coated FSMA ribbons

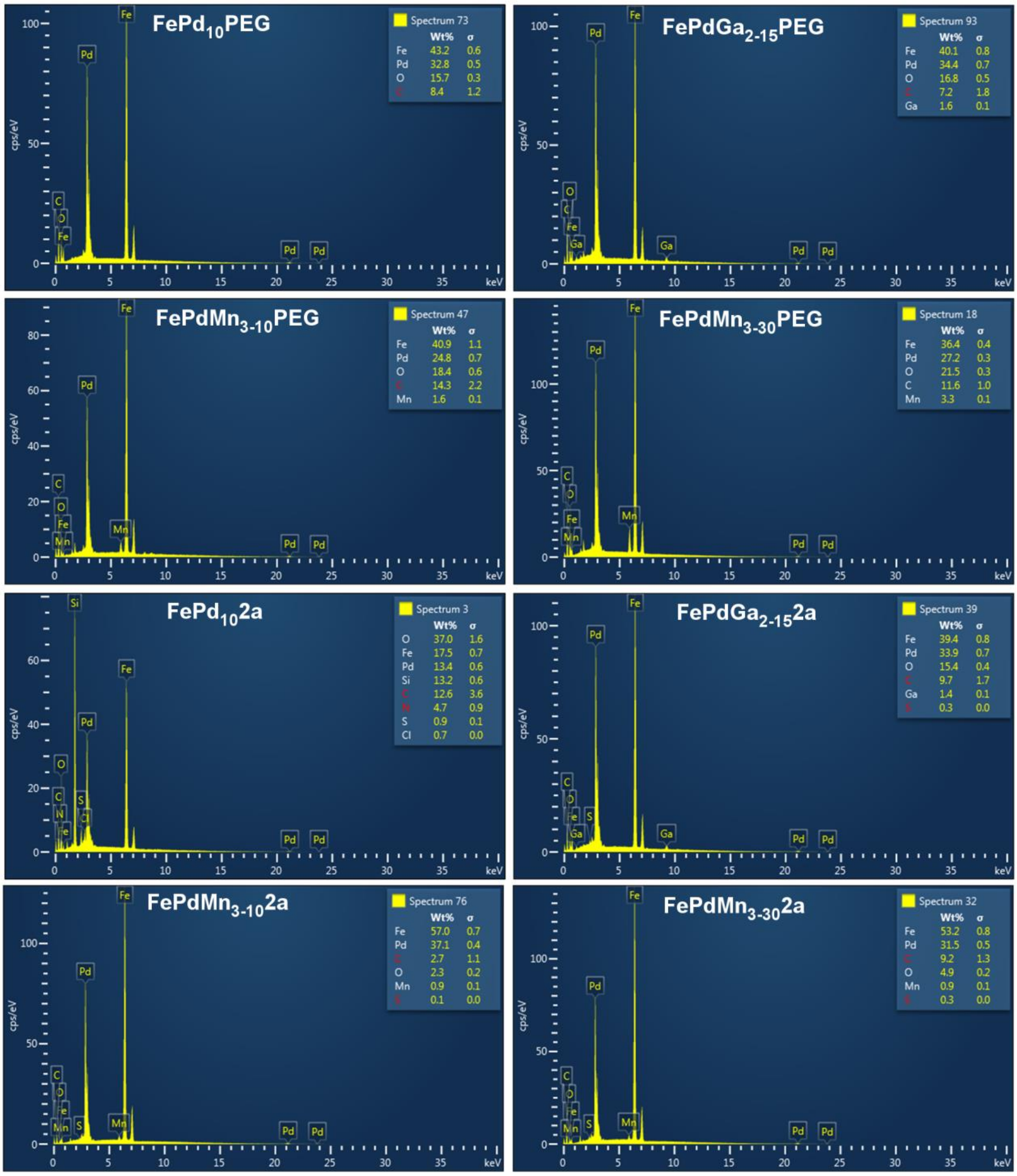

Figure S7: selected EDX spectra of coated FSMA ribbons

Table S1: average PT and APTT values in static and dynamic conditions; normal range: PT = 11,1 – 16,7 s, APTT = 22,4 – 39,5 s

Entry	Sample	PT/INR [s] -static-	APTT [s] -static-	PT/INR [s] -dynamic-	APTT [s] -dynamic-
1	Normal	10.5 / 0.9	34	11.2 / 1.0	34
2	Positive control	11.4 / 0.9	32.7	11.4 / 0.9	32.7
3	$FePd_{10}$	11.2 / 0.9	33.2	13.2 / 1.1	38.2

4	$FePdGa_{2\text{-}15}$	11.2 / 0.9	35.8	14.2 / 1.2	39.8
5	$FePdMn_{3\text{-}10}$	11.2 / 0.9	30.8	11.8 / 0.9	33.8
6	$FePdMn_{3\text{-}30}$	11.2 / 0.9	31.2	11.2 / 0.9	35.2
7	$FePd_{10}PEG$	10.7 / 0.9	38.5	10.7 / 0.9	37.3
8	$FePdGa_{2\text{-}15}PEG$	10.2 / 0.9	24.9	10.6 / 0.9	30.6
9	$FePdMn_{3\text{-}10}PEG$	10.2 / 0.9	22.3	10.3 / 0.9	30.3
10	$FePdMn_{3\text{-}30}PEG$	15.1 / 1.2	31.9	12.1 / 1.1	39.5
11	$FePd_{10}1$	11.7 / 1.0	30.2	11.7 / 0.9	37.3
12	$FePdGa_{2\text{-}15}1$	13.6 / 1.1	39.3	11.2 / 0.9	35.2
13	$FePdMn_{3\text{-}10}1$	12.2 / 1.0	31.7	14.2 / 1.2	39.8
14	$FePdMn_{3\text{-}30}1$	12.5 / 1.1	33.5	11.8 / 0.9	33.8
15	$FePd_{10}2a$	12.5 / 1.0	44.5	12.6 / 1.1	46.2
16	$FePdGa_{2\text{-}15}2a$	14.3 / 1.0	44.7	10.3 / 0.9	47.3
17	$FePdMn_{3\text{-}10}2a$	11.5 / 1.0	40.9	12.1 / 1.1	44.2
18	$FePdMn_{3\text{-}30}2a$	13.6 / 1.0	43.3	11.9 / 1.0	45.1
19	$FePd_{10}2b$	11.9 / 0.9	52.6	11.6 / 0.9	54.9
20	$FePdGa_{2\text{-}15}2b$	12.7 / 1.0	50.6	12.5 / 1.0	57.3
21	$FePdMn_{3\text{-}10}2b$	12.5 / 1.0	47.5	13.1 / 1.0	50.5
22	$FePdMn_{3\text{-}30}2b$	12.4 / 1.0	49.3	11.9 / 0.9	51.2
23	$FePd_{10}2c$	11.5 / 0.9	61.5	11.4 / 0.9	65.4
24	$FePdGa_{2\text{-}15}2c$	14.3 / 1.0	64.5	12.7 / 1.0	65.3
25	$FePdMn_{3\text{-}10}2c$	12.4 / 1.0	49.7	12.5 / 1.0	51.8
26	$FePdMn_{3\text{-}30}2c$	13.1 / 1.0	52.4	12.3 / 1.0	55.4

[1] P.W. Preisler, L. Berger, Preparation of Tetrahydroxyquinone and Rhodizonic Acid Salts from the Product of the Oxidation of Inositol with Nitric Acid, *J. Am. Chem. Soc.*, **1942**, *64*, 67-69.
[2] L.J. Silvestri, R.E. Hurst, L. Simpson, J.M. Settine, Analysis of Sulfate in Complex Carbohydrates, *Anal. Biochem.*, **1982**, *123*, 303-309.
